# High-Performance Porous Supports Based on Hydroxyl-Terminated Polysulfone and CO_2_/CO-Selective Composite Membranes

**DOI:** 10.3390/polym16243453

**Published:** 2024-12-10

**Authors:** Dmitry Matveev, Tatyana Anokhina, Alisa Raeva, Ilya Borisov, Evgenia Grushevenko, Svetlana Khashirova, Alexey Volkov, Stepan Bazhenov, Vladimir Volkov, Anton Maksimov

**Affiliations:** 1 A.V.Topchiev Institute of Petrochemical Synthesis, Russian Academy of Sciences, Leninsky Prospect, 29, 119991 Moscow, Russia; tsanokhina@ips.ac.ru (T.A.); raevaau@ips.ac.ru (A.R.); evgrushevenko@ips.ac.ru (E.G.); avolkov@ips.ac.ru (A.V.); sbazhenov@ips.ac.ru (S.B.); vvvolkov@ips.ac.ru (V.V.); 2 Department of Chemistry, Lomonosov Moscow State University, Leninskie Gory, 1, 119991 Moscow, Russia; max@ips.ac.ru; 3 Progressive Materials and Additive Technologies Center, Kabardino-Balkarian State University, St. Chernyshevsky, 173, 360004 Nalchik, Russia; new_kompozit@mail.ru

**Keywords:** composite membrane, high-performance porous support, gas separation membrane, CO_2_/CO separation, polysulfone, F-containing polysiloxane, polymer synthesis, chemical structure, molecular weight

## Abstract

The scope of this work was to develop a thin-film composite (TFC) membrane for the separation of CO_2_/CO mixtures, which are relevant for many processes of gas processing and gasification of carbon-based feedstock. Special attention was given to the development of highly permeable porous polysulfone (PSF) supports (more than 26,000 GPU for CO_2_) since both the selective and support layers contribute significantly to the overall performance of the TFC membrane. The PSF porous support is widely used in commercial and lab-scale TFC membranes, and its porous structure and other exploitation parameters are set during the non-solvent-induced phase separation (NIPS) process. Since the casting solution properties (e.g., viscosity) and the interactions in a three-component system (polymer, solvent, and non-solvent) play noticeable roles in the NIPS process, polysulfone samples in a wide range of molecular weights (M_w_ = 76,000–122,000 g·mol^−1^) with terminal hydroxyl groups were synthesized for the first time. Commercial PSF with predominantly terminal chlorine groups (Ultrason^®^ S 6010) was used as a reference. The PSF samples were characterized by NMR, DSC, and TGA methods, and the Hansen solubility parameters were calculated. It was found that increasing the ratio of terminal –OH over –Cl groups improved the “solubility” of PSF in N-methyl-2-pyrrolidone (NMP) and water. A direct dependence of the gas permeance of porous supports on the coagulation rate of the casting solution was identified for the first time. It was shown that the use of synthesized PSF (M_w_ = 76,000 g·mol^−1^, M_w_/M_n_ = 3.0, (–OH):(–Cl) ratio of 4.7:1) enabled a porous support with a CO_2_ permeance of 26,700 GPU to be obtained, while the support formed from a commercial PSF Ultrason^®^ S 6010 (M_w_ = 68,000 g·mol^−1^, M_w_/M_n_ = 1.7, (–OH):(–Cl) ratio of 1:1.9) under the same conditions demonstrated 4300 GPU. The siloxane-based materials were used for the selective layer since the thin films based on rubbery polymers do not undergo the same accelerating physical aging as glassy polymers. Two types of materials were screened for the selective layer: synthesized polymethyltrifluoroethylacrylate siloxane-polydecylmethylsiloxane (50F3) copolymer, and polydimethylsiloxane (PDMS). 50F3 siloxane was studied for gas separation applications for the first time. It was shown that the permeance of composite membranes based on high-performance porous supports from the PSF samples synthesized was 3.5 times higher than that from similar composite membranes based on supports from a commercial Ultrason^®^ S 6010 PSF with a permeance value of 4300 GPU for CO_2_. It was found that the enhanced gas permeance of composite membranes based on the highly permeable porous PSF supports developed was observed for both 50F3 polysiloxane and commercial PDMS. At the same time, the CO_2_/CO selectivity of the composite membranes with a 50F3-selective layer (9.1–9.3) is 1.5 times higher than that of composite membranes with a PDMS-selective layer. This makes the F-containing 50F3 polysiloxane a promising polymer for CO_2_/CO separation.

## 1. Introduction

Carbon monoxide (CO) is a valuable gas that finds application in various fields such as the food industry [1], medical industry [2], and metallurgy for recovery of metals from ores [3]. Carbon monoxide is widely used in the chemical industry in the synthesis of various organic compounds such as methanol, phosgene, formic and acetic acids, etc. CO is prepared by various methods, the most common ones being the combustion of carbon and its compounds under oxygen deficiency conditions, reduction in CO_2_ by glowing charcoal, and steam conversion of natural gas to produce synthesis gas. In recent years, the non-thermal plasma technology for CO_2_ conversion has attracted special attention [4]. Unfortunately, CO_2_ is always present in all the systems and has to be removed before the synthesis of fuels and chemical compounds based on CO or synthesis gas [5].

Modern CO_2_/CO separation technologies are based on the following main methods: cryogenic distillation, short-cycle adsorption, absorption, and membrane separation [5]. The advantages of the membrane separation technology over the methods listed above include its compactness, environmental friendliness, relatively low operating cost, continuous separation process, lack of phase transitions (energy efficiency), easy scalability, and simple integration into an existing industrial process [6]. The industrial membrane separation of gases has been developing for over four decades, and the application areas of this technology are expanding continuously [6,7]. Gas separation membranes are mainly manufactured as hollow fibers or flat sheets. Membrane modules based on flat-sheet membranes can be made in a spiral-wound or flat-frame configuration. The membrane modules based on flat-sheet membranes have found commercial use for CO_2_ extraction, e.g., extraction from methane mixtures (Separax membrane, UOP Company, Anaheim, CA, USA; Grace membrane, Kvaerner company, Fornebu, Norway) [6,8]) and from flue gases (Polaris membrane, MTR Company, Newark, NJ, USA; PolyActive membrane, HZG Company, Coburg, Germany) [9]). These membranes are also applied for CO_2_ extraction from natural gases.

An important aspect of membrane efficiency in a real separation process includes both its selectivity and performance [10]. The intensely developing direction in academic research and commercialization of new types of membranes involves the creation of composite membranes with ultra-thin selective layers of micron and nanometer dimensions [11,12]. The most common variant of a composite membrane is a structure comprising a layer of selective, usually non-porous material on the surface of a porous support made of another material. The high interest in composite membranes is explained by a number of advantages. In particular, the selective layer and support can be developed independently, thus making it possible to obtain the required properties for each of the layers [11]. In addition, the use of various selective layer materials (e.g., polydimethylsiloxane (PDMS), polyaminosiloxane, polytrimethylsilylpropyne, cellulose acetate, cellulose nitrate, polyether block amide, polyvinylpyridine, etc. [13]) and porous supports (e.g., polyestersulfone (PES), polysulfone (PSF), polyacrylonitrile (PAN), polyvinylidenefluoride (PVDF), polyphenylene sulfide (PPS), polytetrafluorethylene, polyethyrimide, polyimide, cellulose acetate, polyethylene, etc. [13]) provides a wide range of options for various applications.

Siloxane rubbers, in particular, polydimethylsiloxane (PDMS), are promising for application as a material of selective layers in composite membranes for the separation of CO_2_ and CO gases [5]. In this review, the authors estimated a reasonable range of membrane areas to allow the separation of the two components with sufficiently high recovery of both CO_2_ and CO (about 90% or higher) and good purity (about 90%). The high permeability of PDMS provides the smallest membrane area required, but the separation process is not very efficient in this case [5]. Thus, the problem of increasing the selectivity of highly permeable siloxane membranes arises. One of the approaches to solve this problem can involve a chemical modification of the siloxane chain by incorporating side groups of different natures. It is known that the presence of polar compounds, such as fluorinated groups, in the siloxane structure increases the selectivity for CO_2_ [14]. Previously, we have synthesized fluorinated polysiloxane 50F3 and successfully applied it for pervaporation-based extraction of alcohols from ABE-fermentation mixtures [15].

However, not only the selective layer but also the porous support make a significant contribution to the transport and separation properties of a composite membrane. Recently, it has been shown that the support structure, and hence its performance, contributes significantly to the overall resistance to mass transfer through a composite membrane [12,16,17,18,19,20,21,22]. For example, Park et al., in the “*Science*” journal, pointed out the effect of support resistance on the upper performance limit of a thin-film composite membrane [16]. It was shown that the performance and selectivity limits of the composite membrane, for example, for the CO_2_/N_2_ gas pair, expanded as the performance of the porous support increased for the same thickness of the thin selective layer [16]. In addition, it was shown [17] that as the thickness of the selective layer decreases, the contribution of support resistance to the mass transfer through the composite membrane increases. In this regard, not only the thickness of the selective layer should be reduced, but also the porous structure of the support should be optimized to minimize the resistance to the permeate flow in the development of high-performance composite membranes. Thus, the main requirements for the support include (i) a narrow skin-layer pore size distribution for deposition of a defect-free selective layer and (ii) resistance to solutions from which the selective layer of the composite membrane is applied [13,23].

Polysulfone (PSF) is a well-proven membrane material used in the production of porous filtration membranes and as a support for composite membranes [24,25]. The extensive use of PSF is due to its availability, high temperature resistance, high mechanical strength, and chemical stability [24,25]. PSF is resistant to mineral acids, alkalis, salt solutions, alcohols, aliphatic hydrocarbons, oils, and esters. As a rule, PSF membranes are obtained by the phase separation method. The essence of this approach is to create conditions such that the polymer solution undergoes phase separation and the polymer precipitates to form a solid framework of the polymer membrane, while the liquid phase fills its pores. The implementation of this approach using non-solvent-induced phase separation (NIPS) is the most popular method for producing asymmetric PSF membranes [24,25]. The NIPS technique enables the setting of the porous structure and other parameters for PSF-based membranes [24,25].

The properties of membranes produced by the NIPS method are mainly determined by thermodynamic and kinetic factors. The thermodynamic aspects of phase inversion that are largely determined by the particular polymer/solvent/coagulant system are important for determining the thermodynamically favored phase transitions that can occur in the polymer solution during the membrane formation process [26,27]. At the same time, the kinetics are determined by the rate of the coagulation process [27]. Hence, the kinetic parameters directly affect the properties of the resulting membranes. As shown elsewhere [28] for flat-sheet porous polyamic-acid membranes as an example, an increase in the filtration characteristics occurred with an increase in the coagulation rate.

The NIPS method allows one to obtain supports in a wide range of pore sizes and porosity. Chemical modifications [29,30,31], the incorporation of various fillers into the membrane matrix [32,33,34,35], or the introduction of various additives in coagulation baths [36] are generally used to improve the performance of PSF membranes. For example, in the case of hollow fiber membranes, varying the spinning process parameters makes it possible to control the transport properties of the resulting membranes [37,38]. A new concept for spinning porous PSF fiber supports with an inert bore liquid was suggested in [39]. The new technique made it possible to obtain supports with gas permeance, which was an order of magnitude higher than that of supports reported in the literature.

The properties of the polymer solution, and thus, the properties of the final product, i.e., the support, can be affected by the characteristics of the matrix polymer itself, primarily the molecular weight [40,41,42]. The majority of studies on the application of PSF as a membrane and support material employ commercial grades with similar molecular weights: Udel^®^ P 3500, Solvay, M_w_ = 48,600–50,800 g·mol^−1^ [43,44,45]; Ultrason^®^ S 6010, BASF, M_w_ = 45,000–55,000 g·mol^−1^ [46,47,48]; PSF, Sigma-Aldrich, M_w_ = 35,000 g·mol^−1^ [49,50,51].

At the same time, there are rather few works on varying the molecular weights of polyarylene sulfones [40,42,52,53]. The general trend was that the pore size in the skin layer increased, and the porosity of the membranes decreased with an increase in the polymer molecular weight, resulting in the formation of ultrafiltration membranes with higher strength, higher performance, and a lower retention coefficient. Previously, we have synthesized and studied PSF samples with chlorine and hydroxyl terminal groups [53]. Porous membranes were produced, and the best gas-transport properties were demonstrated by PSF samples with predominantly hydroxyl terminal groups. However, the narrow range of molecular weights of these samples (M_w_ = 57,000–65,000 g·mol^−1^) and low molecular weights limited the options for optimizing the properties of porous supports.

The goal of this work was to synthesize PSF samples with terminal -OH groups in a wider range of molecular weights (M_w_ = 76,000–122,000 g·mol^−1^), to produce flat-sheet asymmetric porous membrane supports and study their morphology, porous structure, and transport properties. A commercial sample of Ultrason^®^ S 6010 PSF (BASF, Ludwigshafen, Germany) was chosen as a comparison polymer. In the present work, 50F3 polysiloxane was studied for the first time as a material for dense films and composite membranes for CO_2_/CO separation applications. In addition, to compare the transport properties of 50F3 with PDMS, composite membranes from a commercially available PDMS (Sylgard 184, Dow Chemical, Midland, MI, USA) were also obtained.

## 2. Materials and Methods

### 2.1. Materials

2,2-Bis(4-hydroxyphenyl)propane (Bisphenol A, 97%), N,N-dimethylacetamide (DMAc) from Acros Organics (Geel, Belgium), 4,4′-dichlorodiphenylsulfone (DCDPS, 99%) from Alfa Aesar (Heysham, UK), and potassium carbonate of “chemically pure grade” from Reachem (Moscow, Russia) were used for the synthesis of PSF samples. DMAc was purified by distillation with calcium hydride and stored with 4-A° molecular sieves. The other reagents were used without additional purification. For F-containing siloxane synthesis polymethylhydrosiloxane (PMHS, M_n_ = 1900 g·mol^−1^, ABCR, Karlsruhe, Germany), trifluoroethylacrylate (98 wt. %, PIM-Invest, Moscow, Russia), toluene (chemically pure grade, Component-Reaktiv, Moscow, Russia), 1-decene (98 wt. %, ABCR, Germany), isooctane (chemically pure grade, Component-Reaktiv, Russia), Karstead catalyst (1,3-divinyl-1,1,3,3,3-tetramethyldisiloxane platinum(0) complex) solution in xylene (Sigma-Aldrich, St. Louis, MO, USA), and vinyl-terminated polydimethylsiloxane (PDMS) (M_n_ = 25,000 g·mol^−1^, Sigma-Aldrich, USA) were used without additional purification.

Commercial reagents, namely the Ultrason^®^ S 6010 (BASF, Germany) and PDMS polymers (Sylgard 184, Dow Chemical, Midland, MI, USA), were used in this work.

To prepare casting solutions, N-methyl-2-pyrrolidone (NMP, Acros Organics, Geel, Belgium) was used as the solvent, while polyethylene glycol with an average molecular weight of 400 g·mol^−1^ (PEG-400, Sigma-Aldrich, Overijse, Belgium) was used as the pore-forming additive.

### 2.2. PSF Synthesis

**Figure 1:**
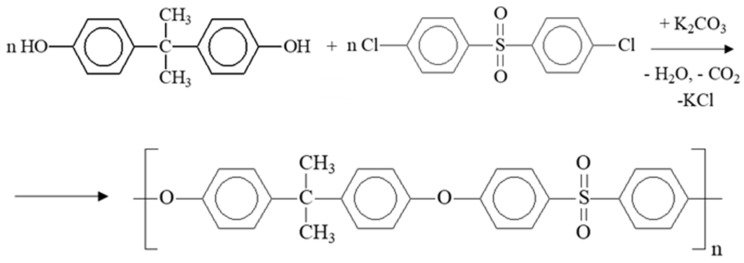
Scheme of PSF synthesis.

PSF samples were synthesized by high-temperature polycondensation by the nucleophilic substitution mechanism using various ratios of Bisphenol A and DCDPS monomers. The synthesis scheme is shown in Figure 1.

The synthesis was carried out in the presence of potassium carbonate as the alkaline agent to convert the hydroxyl groups of Bisphenol A to reactive phenolate groups. Water was continuously removed from the reaction zone by distillation. Polycondensation was carried out in a glass reactor system equipped with a top-drive stirrer, thermocouple, capillary for inert gas supply, Dean–Stark trap, and reflux condenser. The synthesis was performed in DMAc medium using Bisphenol A with DCDPS molar ratios of 1: (1.05–1.11). The synthesis temperature was kept at 166 °C, i.e., the boiling point of DMAc. The reaction solution was then cooled to 30 °C, and the polymer was precipitated by sputtering into distilled water acidified with hydrochloric acid. The polymer was filtered off and washed repeatedly with hot distilled water to remove low molecular weight reaction products and the solvent. The polymer powder was dried in a vacuum oven at 120 °C for 12 h. The molecular weight of the resulting PSF samples was controlled by the ratio of the starting monomers according to the nonequivalence rule.

### 2.3. Study of the Properties of the PSFs Synthesized

#### 2.3.1. Gel Permeation Chromatography (GPC)

Gel permeation chromatography of the PSF samples was performed on a Waters system with a differential refractometer (Chromatopack Microgel-5, chloroform as the eluent, flow rate 1 mL/min). The molecular weights M_w_, M_n_, and polydispersity M_w_/M_n_ were calculated according to the standard procedure with respect to monodisperse polystyrene standards.

#### 2.3.2. Nuclear Magnetic Resonance (NMR)

High-resolution ^1^H and ^13^C NMR spectra were obtained on a Bruker AVANCE III HD 400 NMR spectrometer in CDCl_3_ by the standard procedure.

#### 2.3.3. Differential Scanning Calorimetry (DSC)

DSC analysis was performed on a Perkin Elmer DSC 4000 instrument in a nitrogen atmosphere in the temperature range from 25 to 250 °C. The scanning rate during heating was 10 °C/min. The values of the glass transition temperature obtained during the second heating of the sample were taken as the analysis results.

#### 2.3.4. Thermogravimetric Analysis (TGA)

Thermogravimetric analysis of the polymers was carried out on a Perkin Elmer TGA 4000 derivatograph (Waltham, MA, USA) in the air. The scanning rate during heating was 10 °C/min. The studies were carried out in the temperature range from 30 to 750 °C.

#### 2.3.5. Calculation of the Hansen Solubility Parameters

The solubility parameters of the synthesized and commercial PSFs were calculated using the Hoftyzer–van Krevelin group contribution method [54]. Equations (1)–(3) were used to calculate *δ_d_*, *δ_p_*, and *δ_h_* parameters characterizing the dispersion and polar interactions and the interactions due to formation of hydrogen bonds, respectively:

(1) $$\delta_{d}=\frac{\sum F_{di}}{V}$$

(2) $$\delta_{p}=\frac{\sqrt{\sum F_{pi}^{2}}}{V}$$

(3) $$\delta_{h}=\sqrt{\frac{\sum F_{hi}}{V}}$$

where *F_di_* is the group contribution of the dispersion component, *F_pi_* is the group contribution of the polar component, *E_hi_* is the group contribution of the energy of hydrogen bonds in the PSF macromolecule, and *V* is the molar volume of the PSF macromolecule. The ratio of terminal groups and the number of monomer units in the PSF macromolecule were taken from the analysis of the NMR spectra. The group contributions of the solubility parameter components for PSF are presented in Table 1.

The total solubility parameter *δ* was calculated using Equation (4):

(4) $$\delta^{2}=\delta_{d}^{2}+\delta_{p}^{2}+\delta_{h}^{2}\;$$

The Hansen Solubility Parameter distances (HSP-distances) and the values determining the polymer “solubility” in solvent ∆*δ_P-S_* and non-solvent ∆*δ_P-NS_*, respectively, were calculated by Equations (5) and (6):

(5) $$∆\delta_{P-S}=[{\left(\delta_{d,P}-\delta_{d,S}\right)}^{2}+{\left(\delta_{p,P}-\delta_{p,S}\right)}^{2}+{\left(\delta_{h,P}-\delta_{h,S}\right)}^{2}{]}^{1/2}$$

(6) $$∆\delta_{P-NS}=[{\left(\delta_{d,P}-\delta_{d,NS}\right)}^{2}+{\left(\delta_{p,P}-\delta_{p,NS}\right)}^{2}+{\left(\delta_{h,P}-\delta_{h,NS}\right)}^{2}{]}^{1/2}$$

where the *P* index corresponds to the polymer solubility parameters, the *S* index to the solvent parameters, and the *NS* index to the non-solvent parameters. These parameters were calculated for the PSF-NMP and PSF-water pairs.

### 2.4. Study of Dense Films Prepared from the PSFs Synthesized

#### 2.4.1. Preparation of PSF Films

Dense films were obtained from PSF/NMP solutions with a polymer concentration of 20 wt. %. The films were prepared by casting the polymer solution onto a glass support followed by incubation in an oven for 14 days at 50 °C and then for 24 h at 230 °C, which is above the glass transition temperature of the polymer (~190 °C). The thickness of the films obtained was 170 ± 20 μm.

#### 2.4.2. Measurement of the Contact Angles of PSF Films

The contact angles were measured by the “sessile drop” technique using a goniometer (PRC OpenScience Ltd., Krasnogorsk, Russia). Data acquisition and subsequent digital processing of water droplet images for calculating the contact angles by the Yang–Laplace equation were performed using the DropShape v.1.0 program. The temperature of the measurements was 25 °C.

#### 2.4.3. Study of the Mechanical Properties of PSF Films

The mechanical properties of polymer films (Young’s modulus and tensile strength (MPa) and elongation at break (%)) were determined on an I1140M tensile machine (LLC “TOCHPRIBOR-KB”, Ivanovo, Russia) with a plunger movement speed of 10 mm/min. Films 4–5 mm wide and 60 mm long were used as the samples. The Young’s modulus values were determined as the slope of the initial (linear) section of the stress–strain diagram.

### 2.5. Study of the Properties of Casting Solutions

#### 2.5.1. Preparation of Polymer Solutions

The PSF samples were used to prepare two-component solutions in NMP and three-component solutions in NMP with the addition of the PEG-400 pore-forming additive. The concentration of PSF in all the solutions was 21 wt. %. In the case of three-component solutions, the concentration of PEG-400 was 30 wt. %. Similar polymer solutions were prepared from commercial PSF Ultrason^®^ S 6010 (BASF, Germany), which is widely used for the fabrication of porous asymmetric membranes [46,47,48]. The casting solutions were stirred at room temperature until complete homogenization (for at least 16 h).

#### 2.5.2. Study of Phase Inversion Kinetics

The phase inversion kinetics for polymer solutions of PSF/NMP (21/79 wt. %) were studied using an original technique for measuring the coagulation rate in a “limited” layer of the polymer solution [28]. This technique allows one to simulate the formation of a flat-sheet polymer membrane with a given thickness and visualize the structure formation in an asymmetric membrane. Distilled water was used as the coagulant. The phase inversion kinetics was measured by determining the coagulation rate of a polymer solution layer with a given thickness (200–300 μm in this work). The coagulation rate was calculated as the ratio of the total thickness of the polymer layer (μm) to the time of its coagulation (s). The results were averaged over three measurements for each polymer solution.

#### 2.5.3. Determination of Dynamic Viscosity

Dynamic viscosity was measured with a MCR 72 rotary rheometer (Anton Paar, Graz, Austria) equipped with a CP60-0.5 cone-plane measuring unit and temperature control devices. The polymer solution was kept at 23 ± 0.1 °C for 5 min in the measuring cell. The shear rate was 10 1/s.

### 2.6. Study of the Properties of Porous PSF Supports

#### 2.6.1. Preparation of Porous PSF Supports

Porous asymmetric supports were prepared by applying PSF/NMP/PEG-400 casting solutions (21/49/30 wt. %) based on the synthesized and commercial (Ultrason^®^ S 6010) PSF samples as a thin layer on glass using a casting knife with a gap thickness of 200 μm. After application, the polymer solution layer was deposited in distilled water. The flat-sheet support samples were then washed sequentially in distilled water for at least 17 h, in ethanol for 2 h, and in n-hexane for 2 h, and air-dried at room temperature.

#### 2.6.2. Study of the Gas-Transportation Properties of Porous Supports

The gas-transport properties of porous PSF supports for individual gases, i.e., helium, nitrogen, and carbon dioxide, were studied by the volumetric method. The differences in the molecular weights of these gases allowed us to reliably determine the occurrence of the Knudsen flow regime by analyzing the values of ideal selectivities, i.e., ratios of permeance for individual gases. The volume of gas flowing through the membrane was determined using a Dry Gas Meter (Shinagawa, Japan). Gas permeance measurements were performed at room temperature (23 ± 2 °C) at transmembrane pressures ranging from 0.5 to 2 bar, while the permeate pressure was kept constant at 1 bar.

The permeance was calculated using Equation (7):

(7) $$\frac{P}{l}=\frac{Q}{p\cdot S}$$

where (*P*/*l*) is the permeance of an individual gas, m^3^/(m^2^h atm); *Q* is the volume flow rate of the gas that passed through the membrane, m^3^/h; *p* is the transmembrane pressure, atm; and *S* is the membrane surface area, m^2^.

Ideal selectivity *α* was calculated using the Equation (8):

(8) $$\alpha =\frac{\left(P_{1}/l\right)}{\left(P_{2}/l\right)}=\frac{P_{1}}{P_{2}}$$

#### 2.6.3. Pore Size Measurement

The diameters of the largest pores dmax in flat-sheet supports made of PSF samples were determined using a bubble point test. Galwick (P.M.I., Ithaca, NY, USA), a wetting agent with a surface tension of 15.9 mN/m, was employed in the experiment.

### 2.7. Study of Composite Membranes

#### 2.7.1. Synthesis of Polymethyltrifluoroethylacrylatesiloxane–Polydecylmethylsiloxane Copolymer (50F3)

The polymethyltrifluoroethylacrylatesiloxane–polydecylmethylsiloxane copolymer (50F3) was synthesized by hydrosilylation in the presence of the Karstedt catalyst according to the one-step procedure suggested previously [55]. Polymethylhydrosiloxane was mixed with a mixture of a 15 wt. % solution of trifluoroethylacrylate in toluene and a 15 wt. % solution of 1-decene in isooctane at a 1:1 molar ratio of Karstead catalyst. The resulting mixture was stirred for 2 h at 60 °C. To cross-link the polymer, a 10 wt. % PDMS solution in isooctane was added to the reaction mixture, and the mixture was stirred at 60 °C for 1 h. At the final stage of the synthesis, 3 wt. % PMHS solution was added to reach the PMHS:PDMS molar ratio of 0.16. The resulting polymer solution was brought to a viscosity of 36 mPa s and fixed with dimethyl maleate as a hydrosilylation inhibitor. The viscosity of the solutions was measured using a Brookfield DV2T-RV viscometer at 25 °C. To obtain composite membranes, the polymer solution was diluted with isooctane to a concentration of 10 wt. %.

#### 2.7.2. Preparation of Composite Membranes

To apply selective layers on the support, 50F3 and Sylgard 184 were used. The composite membranes were prepared from a 10 wt. % solution of 50F3 and Sylgard 184 in isooctane by centrifugation on a SpinNXG-P1A coater (Apex Instruments, Kolkata, India). The system was equipped with a foreline pump to support fixing. Porous PSF supports were attached to a flat glass plate using adhesive tape. The polymer solution (1 mL) was fed from a pipette to the center of the rotating support. The rotation speed was 1000 rpm, as determined in the previous study [56].

#### 2.7.3. Study of the Gas-Transport Properties of Porous Supports

**Figure 2:**
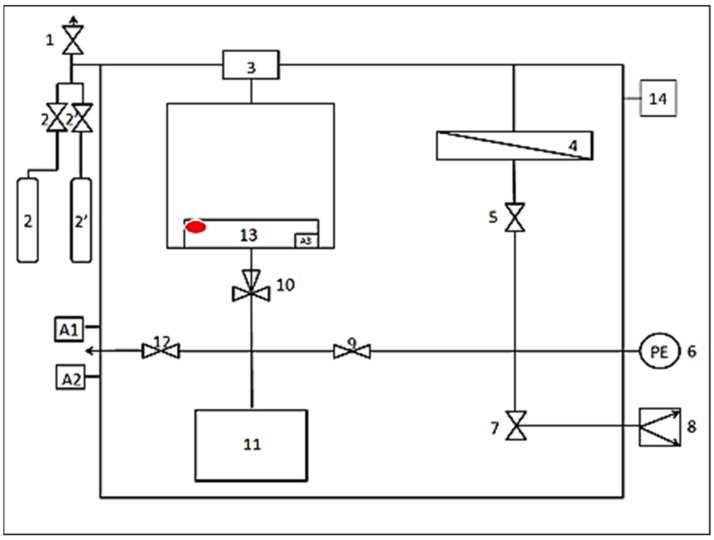
Scheme of the device for measuring gas permeance of composite membranes. Legend: 1,2,2′,5,7,9,12,14—shut-off valves; 3—pressure regulator; 4—membrane cell; 6—pressure sensor; 8—vacuum pump; 10—three-way valve; 11—buffer volume tank; 13—pulse counter combined with the pressure regulator. A1—power supply to the unit; A2—switching of the vacuum pump; A3—switch for setting the overpressure value.

The gas-transport properties of the porous PSF supports were studied for individual gases, CO_2_ and CO, using the device schematically shown in Figure 2.

A membrane to be studied was installed into membrane cell 4. Using pressure regulator 3 and vacuum pump 8, a pressure drop Δ*p* was created across the diaphragm. The time *τ* required for the pressure in the sub-membrane space with volume *V* to change from *p*_1_ to *p*_2_ was measured by pulse counter 13. The gas permeance of the composite membrane *P*/*l* was calculated from the values of *τ*, transmembrane pressure Δ*p*, and membrane area *S* using Equation (9).

(9) $$P/l=\frac{V\cdot (\;p_{2}-p_{1})\;}{∆p\cdot S\cdot \tau }$$

The membrane selectivity of CO_2_/CO was calculated using Formula (8).

### 2.8. Scanning Electron Microscopy (SEM)

The geometry and morphology of porous PSF supports and composite membranes were studied by SEM using a Hitachi Tabletop TM 3030 Plus microscope with a highly sensitive low-vacuum secondary electron detector (Hitachi High Technologies Corporation, Tokyo, Japan). Membrane cross-sections were obtained in liquid nitrogen and then a gold layer was deposited on them using a DSR-1 sprayer (NSC, Tehran, Iran). The thickness of the gold film layer varied between 50 and 100 Å. A Bruker Quantax 70 EDS system was used to analyze the elemental composition by energy-dispersive X-ray spectroscopy (EDX) analysis.

## 3. Results

### 3.1. Chemical Structure and Properties of PSF Samples and Their Solutions

#### 3.1.1. Structure and Molecular Weight of PSF Samples

The synthesized PSF samples were characterized by ^1^H and ^13^C NMR spectroscopy. The samples were dissolved in CDCl3.

**Figure 3:**
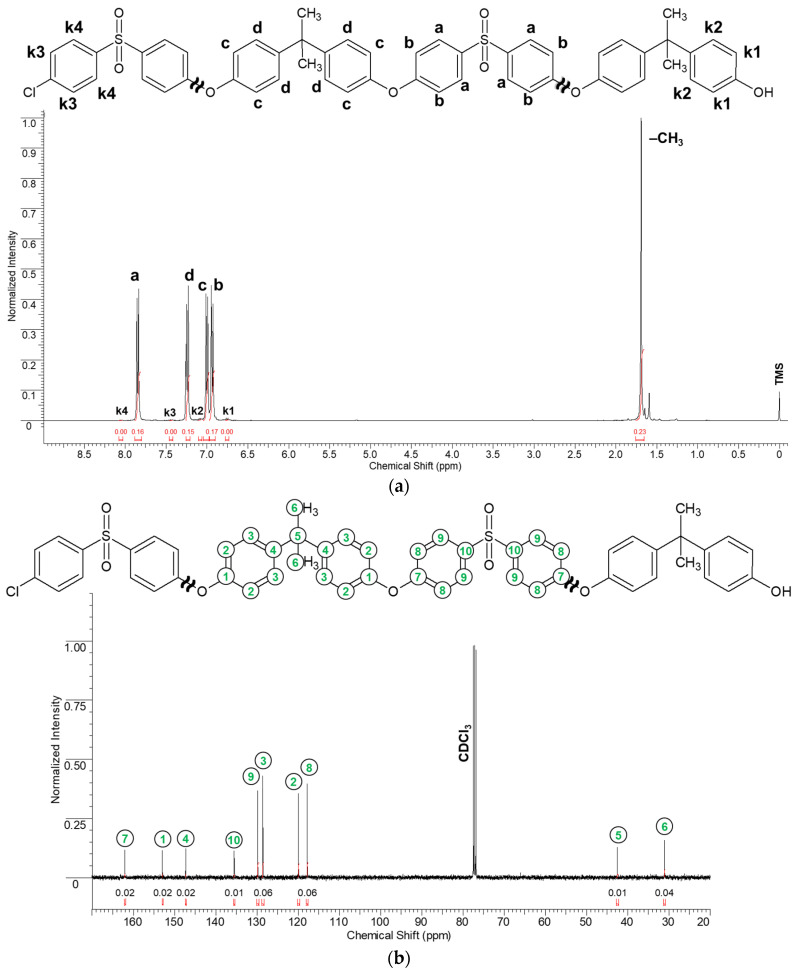
^1^H (**a**) and ^13^C (**b**) NMR spectra of PSF-1 sample.

Figure 3a illustrates the ^1^H NMR spectrum of the PSF-1 sample as an example. The doublet peaks of chemical shifts δ = {7.84 and 7.86 ppm} H-a (4H, d), δ = {6.92 and 6.94 ppm} H-b (4H, d), δ = {6.99 and 7.01 ppm} H-c (4H, d), and δ = {7.23 and 7.25 ppm} H-d (4H, d) correspond to the protons located in the aromatic rings of the main polymer chain. The singlet signal δ = 1.69 ppm (6H, s) was attributed to the protons of the methyl groups of the main chain. The doublet peaks δ = {6.74 and 6.76 ppm} (2H, d) and δ = {7.07 and 7.09 ppm} (2H, d) correspond to the protons of the terminal aromatic rings bound to the terminal hydroxyl groups. The doublet signals δ = {7.44 and 7.47 ppm} (2H, d) and δ = {8.03 and 8.06 ppm} (2H, d) are assigned to the protons of the aromatic rings bound to the terminal chlorine groups.

The ^13^C NMR spectrum of the PSF-1 sample is shown in Figure 3b. Peaks of chemical shift δ = {42.5 and 31.1 ppm} indicate the presence of a quaternary carbon atom C-5 and CH_3_ groups C–6 in the monomer unit. The peaks of chemical shift values δ = {162.1, 153, 147.3 and 135.5 ppm} correspond to carbon atoms in the aromatic ring C-7, C-1, C-4 and C-10, respectively. Two peaks in the region of the chemical shift δ = 129 ppm were attributed to the carbon atoms in the aromatic ring C-3 and C-9, located in the ortho position with respect to the sulfonic and isopropylidene groups. Two peaks in the region of the chemical shift δ = 120 ppm correspond to the carbon atoms in the aromatic ring C-8 and C-2, located in the ortho position with respect to the functional ether group. It was not possible to identify any terminal groups in the samples of PSF by analyzing carbon nuclear magnetic resonance spectra.

^1^H NMR spectroscopy confirmed the presence of predominantly terminal hydroxyl groups in the structure of PSF in the synthesized samples. The ratios of the -OH and -Cl terminal groups in the PSFs were also calculated (Table 2). The ratios were quantitatively determined by comparing the integral areas of the k1 and k3 signals corresponding to the protons in the aromatic rings bound to the terminal hydroxyl and chlorine groups, respectively (Figure 3). It is noteworthy that the commercial PSF Ultrason^®^ S 6010 has predominantly chloride terminal groups (Table 2). Based on the spectral analysis results, the number of monomer units in the polymer chain of the synthesized PSF samples was estimated by calculating the ratio of integral areas of signals of protons of phenylene rings within and at the ends of the polymer chain normalized to one proton. The average molecular weight of the polymer was calculated as the product of the number of monomer units in the polymer chain by the molecular weight of one monomeric link. The results are summarized in Table 2.

The study of molecular weight characteristics by the GPC method showed that the synthesis of PSF with the blocking of terminal groups results in polymers with unimodal molecular weight distribution. Table 2 shows the weight-average M_w_ and number-average M_n_ molecular weights and M_w_/M_n_ polydispersity ratio determined by the GPC method. The synthesized PSF samples have a wide range of molecular weights (M_w_ = 76,000–122,000 g·mol^−1^). The polydispersity coefficient M_w_/M_n_ for all samples has close values within 2.8–3.1. It is noteworthy that the M_n_ and M_NMR_ molecular weights determined by the GPC and NMR methods, respectively, match only qualitatively. Their values differ by 60–80% (see Table 2). This may be due to the high error in the estimation of the number of terminal groups with respect to internal groups in the polymer chain by the NMR method. This is especially true for polymers with high molecular weight where the signal intensity from the terminal links is relatively low [42].

Analysis of the data in Table 2 shows that the synthesized PSF can be divided into three groups based on the quantitative ratio of terminal groups and molecular weight:

(I) PSF-1 and PSF-3 with an (-OH):(-Cl) ratio of 2.4: 1 and molecular weights M_w_ of 122,000 and 100,000 g·mol^−1^, respectively;
(II) PSF-2 and PSF-4 with an (-OH):(-Cl) ratio of (3.0–3.1): 1 and molecular weights M_w_ of 111,000 and 97,000 g·mol^−1^, respectively;
(III) PSF-5 with an (-OH):(-Cl) ratio of 4.7: 1 and a molecular weight M_w_ of 76,000 g·mol^−1^.

The study of the thermal properties by the TGA method showed that the thermal stability of the synthesized PSFs increases with an increase in molecular weight (Table S1, Supplementary Materials). A regular increase in the glass transition temperature was also observed (Figure S1 Supplementary Materials).

#### 3.1.2. Mechanical Properties

**Figure 4:**
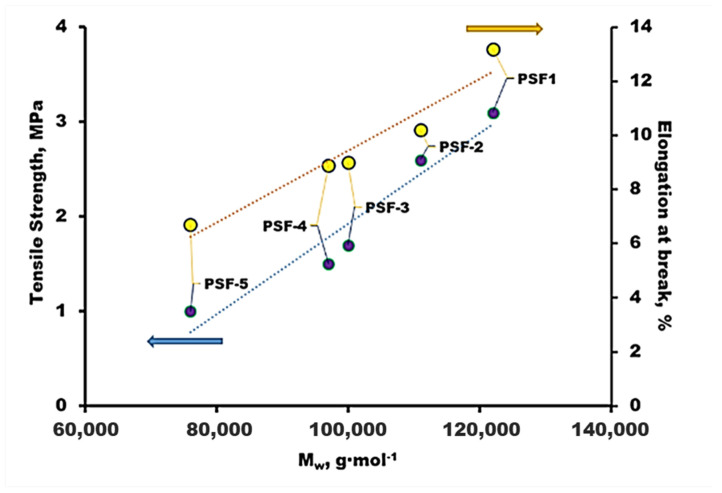
Mechanical properties of dense films made of the PSFs synthesized.

The dependences of mechanical characteristics of films (tensile strength and relative elongation at break) made of synthesized PSF samples on the polymer molecular weight are shown in Figure 4. As one would expect, an increase in the molecular weight of PSF results in an increase in both tensile strength and relative elongation. It can be seen that both dependencies are approximated well enough by nearly linear plots in the presented coordinates. The tensile strength increases approximately 3-fold from 1.0 MPa (PSF-5, M_w_ = 76,000 g·mol^−1^) to 3.1 MPa (PSF-1, M_w_ = 122,000 g·mol^−1^). The relative elongation increases about 2-fold from 6.7% (PSF-5) to 13.2% (PSF-1). Young’s modulus was also determined for the synthesized PSF films. Its value for all the samples is in the range of 160–190 MPa.

#### 3.1.3. Contact Angles

**Figure 5:**
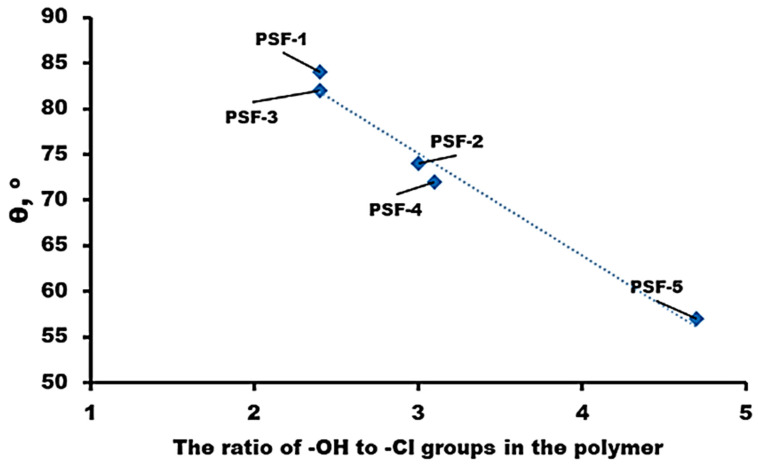
Dependence of the water contact angle of PSF films on the ratio of (-OH):(-Cl) groups in the polymer.

Contact angles were measured for the PSF films using distilled water; the results are given in Figure 5. It can be seen that an increase in the ratio of terminal hydroxyl groups in the polymer from 2.4:1 to 4.7:1 leads to a significant increase in the hydrophilicity of the films obtained: the contact angle decreases from 84° (PSF-1) to 56° (PSF-5).

It should be emphasized that the films from the PSF samples synthesized in this work have higher hydrophilicity than the commercial PSF samples whose water contact angles are 85–104° [57,58,59]. This is due to the fact that the ratio of terminal chlorine groups prevails in the commercial PSF samples.

#### 3.1.4. Solubility Parameters of PSF Samples

Hansen solubility parameters for the PSF samples were calculated using the group contribution method. For commercial PSF, it was assumed that the Ultrason^®^ S 6010 had the same structure of the main chain as the synthesized PSF samples. The ratio of terminal groups (-OH):(-Cl) and the number of monomer units (and, therefore, M_n_) in the polymers were taken from the analysis of the NMR spectra (Table 2).

The calculated *δ_d_*, *δ_p_*, and *δ_h_* parameters are presented in Table 3. It can be seen that the nature of terminal groups in the polymers under study nearly does not affect the dispersion and polar interactions. At the same time, for the synthesized PSF samples, one can note a weak tendency for an increase in the *δ_h_* parameter, which characterizes the interaction due to hydrogen bonds, with an increase in the ratio of terminal groups (-OH):(-Cl) from 2.4: 1 to 4.7: 1. The lowest value of the *δ_h_* = 5.84 (MJ/m^3^)^1/2^ parameter is observed for the commercial PSF Ultrason^®^ S 6010 with the lowest ratio of terminal groups (-OH):(-Cl) = 1: 1.9. The values of ∆*δ_P-S_* and ∆*δ_P-NS_* characterizing the “solubility” of PSF samples in NMP and water, respectively, were also calculated. It is known that the smaller the Δ*δ_P-S_* value, the higher the solvent quality for a given polymer [60]. As can be seen from Table 3, NMP is an almost equally good solvent for all the PSFs, including the commercial one, whereas water is a non-solvent (coagulant) for all the samples.

**Figure 6:**
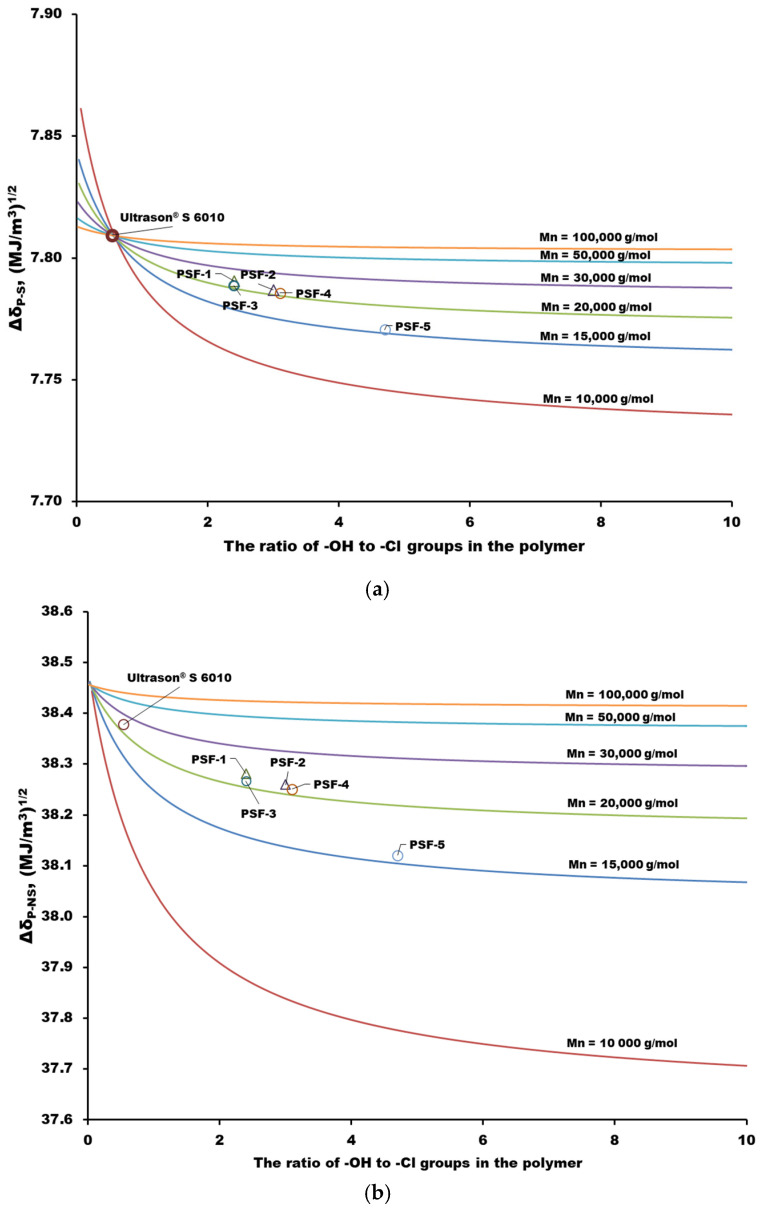
Calculated dependencies of the HSP distance for PSF in NMP (**a**) and water (**b**) on the ratio of hydroxyl groups in the polymer.

The HSP distances in the PSF-NMP and PSF water systems as a function of the ratio of chlorine and hydroxyl terminal groups were calculated for polymers with various numbers of monomer units in the chain corresponding to molecular weights M_n_ = 10,000–100,000 g·mol^−1^. The resulting curves are presented in Figure 6. As can be seen, the solubility of PSF in NMP increases with an increase in the ratio of terminal -OH groups. The polymer has the lowest solubility in NMP in the case of the maximum ratio of terminal chlorine groups in PSF (Figure 6a). A similar dependence of the HSP distance on the ratio of terminal groups is observed in the PSF water system (Figure 6b). It also follows from the curves that the effect of the terminal groups on the solubility of PSF decreases regularly with the growth of the main polymer chain (Figure 6a,b). The latter conclusion confirms the lack of significant differences between the ∆*δ_P-S_* and ∆*δ_P-NS_* values for all PSF samples (Table 3).

#### 3.1.5. Phase Separation Kinetics

The kinetics of phase separation of two- and three-component solutions was studied for PSF samples using the original technique of measuring the coagulation rate in a “limited” layer of a polymer solution [28]. In fact, the movement rate of the phase separation front (coagulation rate, µm/s) was measured using a 200–300 µm thick polymer solution in contact with water. The study was carried out at 25 °C. The PSF concentration in all the solutions studied was 21 wt. %, while the concentration of PEG-400 in the three-component solutions was 30 wt. %. The measured coagulation rates are presented in Table 4; the ratios of PSF terminal groups are given for convenience. To compare the phase separation kinetics of polymer solutions, the average coagulation rate was estimated for the same thickness of the solution layer equal to 200 μm.

An Important conclusion from Table 4 is that the coagulation rate depends significantly, first of all, on the ratio of the terminal hydroxyl and chlorine groups. For example, the PSF/NMP solution based on PSF-5 shows the highest coagulation rate (11.2 µm/s). This polymer has the highest ratio of terminal hydroxyl groups, (-OH):(-Cl) = 4.7: 1. As the ratio of hydroxyl groups decreases, coagulation slows down. The PSF/NMP solution with the commercial PSF Ultrason^®^ S 6010 demonstrates the smallest coagulation rate.

Moreover, the dependence on the polymer’s molecular weight is also an expected result. In fact, a comparison of PSF-1 with PSF-3 and PSF-2 with PSF-4 samples with the same terminal group ratios confirms that the lower molecular weights of PSF-3 and PSF-4 compared to PSF-1 and PSF-2, respectively, result in an increase in the coagulation rate. For example, the coagulation rates of PSF-1 (122,000 g·mol^−1^) and PSF-3 (100,000 g·mol^−1^) samples increase from 4.6 to 4.9 µm/s. This phenomenon may be attributed to the impact of molecular weight on the dynamic viscosity of casting solutions (Figure S2 Supplementary Materials).

It should be noted that an addition of PEG-400 to the casting solution favors an increase in the coagulation rate of polymer solutions. In fact, as follows from Table 4, the addition of 30 wt. % PEG-400 increases the coagulation rate by about 20% for all the PSF samples. Flat-sheet membranes were obtained using the PSF/NMP/PEG-400 casting solution with the (21/49/30 wt. %) composition.

### 3.2. Properties of Porous Supports

#### 3.2.1. Morphology of Porous Supports

**Figure 7:**
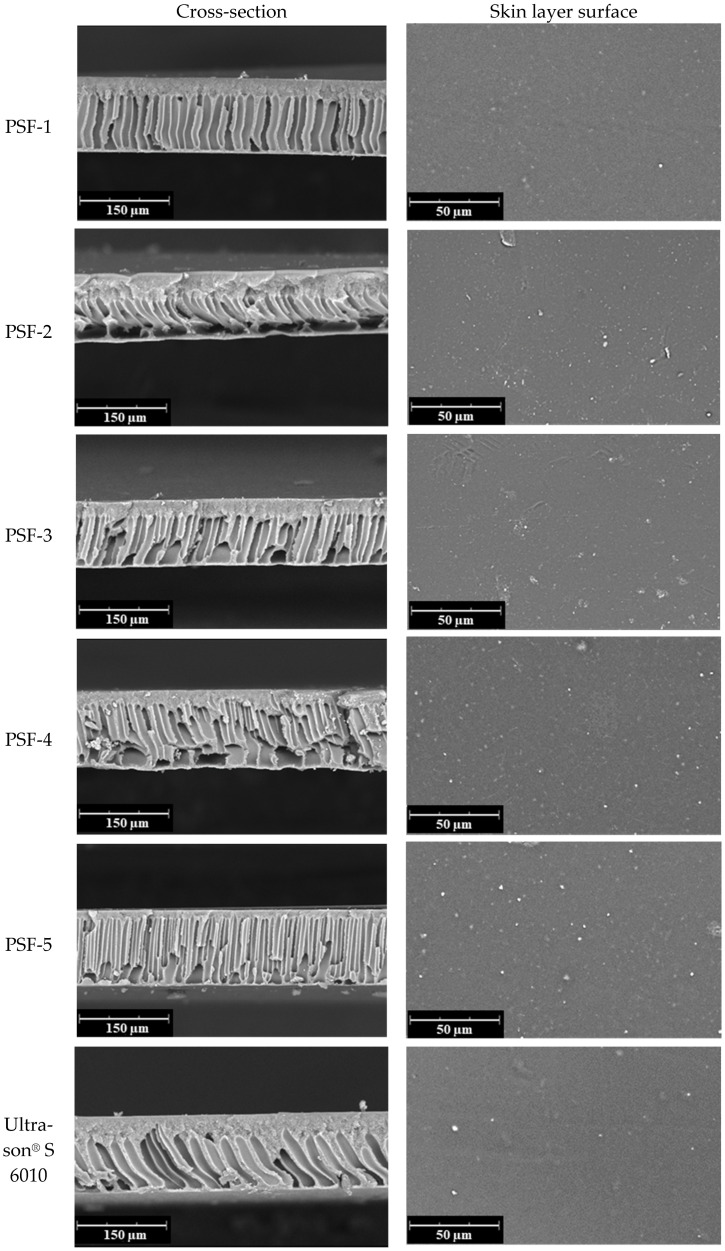
SEM microphotographs of cross-section and skin layer surfaces of porous PSF supports.

The supports obtained were studied by scanning electron microscopy (SEM). Figure 7 shows the cross-section and skin layer surface microphotographs of porous PSF supports. It can be seen that the synthesized and commercial PSF supports have a similar morphology comprising an upper thin porous skin layer, a transition layer with a spongy structure, and an inner drainage layer with large finger-like macrovoids. The developed porous supports exhibit an asymmetric structure, which is typical of porous PSF supports obtained using the NIPS method with water used as a coagulant [39,45]. Finger-like macrovoids can occur as a result of a high phase separation rate, as well as due to thermal effects generated during the mixing of the solvent and coagulant (NMP and water) [27]. It should be noted that there is a trend for the synthesized PSF samples to exhibit a decrease in the thickness of the transition layer with a spongy structure, ranging from 30 μm (PSF-1) to 10 μm (PSF-5). This may be attributed to a reduction in the viscosity of the casting solution (see Table 2), as viscosity can influence the morphology of polymeric membranes [62]. The average thickness of all the supports is 110–120 μm (Table 5).

#### 3.2.2. Gas-Transport Properties

The gas-transport properties of the supports were studied for individual He, N_2_, and CO_2_ gases. The difference in the molecular weight of these gases allows the existence of the Knudsen flow regime (α = 3.3 for the He/CO_2_ gas pair, α = 2.6 for the He/N_2_ gas pair) to be estimated according to the values of ideal selectivities. The realization of the Knudsen regime of gas flow indicates the absence of defects, which is important for depositing thin selective layers of composite membranes.

The gas-transport properties of porous supports are summarized in Table 5. It can be seen that the highest permeance (e.g., 26,700 GPU for CO_2_) is demonstrated by the support made of the PSF-5 polymer that has the lowest molecular weight and the highest content of terminal hydroxyl groups. High values of transport properties are also observed for the porous supports made of the PSF-2 and PSF-4 polymers, whose CO_2_ permeance is 18,900 and 22,300 GPU, respectively. It should be noted that the lowest permeance is demonstrated by the porous support made of commercial PSF Ultrason^®^ S 6010 (4300 GPU for CO_2_). It should also be noted that the ideal selectivities for the He/N_2_ and He/CO_2_ gas pairs for all the supports have close values α (He/N_2_) = 2.3–2.5 and α (He/CO_2_) = 2.9–3.2, indicating that the regime of gas flow through the porous structure of the supports approximates the Knudsen regime. At the same time, the He/CO_2_ selectivity tends to decrease with an increase in the size of the largest pore d_max_, i.e., a slight increase in the contribution of Poiseuille flow to Knudsen flow is observed.

**Figure 8:**
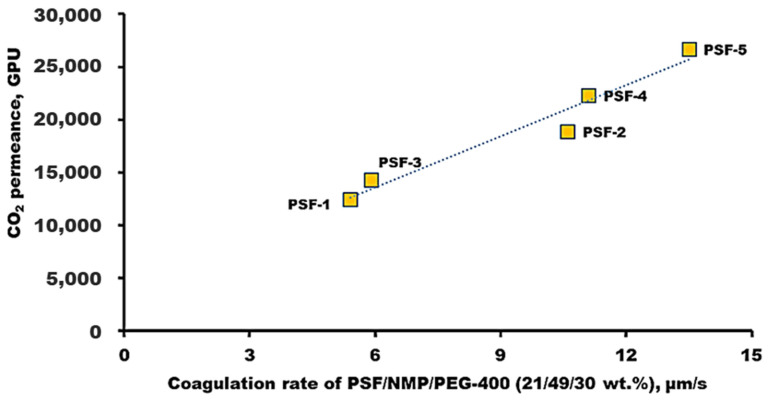
Dependence of CO_2_ permeance of porous PSF supports on the coagulation rate of PSF/NMP/PEG-400 casting solutions (21/49/30 wt. %).

The dependence of CO_2_ permeance of porous PSF supports on the coagulation rate of PSF/NMP/PEG-400 casting solutions (21/49/30 wt. %) is shown in Figure 8. The permeance of the porous PSF supports obtained increases with an increase in the coagulation rate of the casting solutions and (-OH):(-Cl) ratio. Apparently, a high coagulation rate favors the formation of a more porous skin layer in the membranes. A similar dependence was obtained in another study [28], where an increase in the permeability of a water-rich isobutanol solution of porous polyamic acid membranes was observed with an increase in the coagulation rate of the casting solutions.

Table 6 presents a comparison of the transport properties of the PSF supports developed with data reported in the literature for flat-sheet porous membranes used for the subsequent production of gas separation composite membranes. It is noteworthy that in many cases, only the gas-transport properties of composite membranes are presented in the literature without considering the transport properties of porous supports. As can be seen from Table 6, the PSF supports developed in the present work are superior to their PSF analogs in terms of gas-transport properties [21,63]. In addition, they are also not inferior to the properties of flat-sheet porous supports made of other polymers (PAN, PVDF, PES).

### 3.3. Composite Membranes

Taking into account the combination of transport and mechanical properties of the supports developed in this work (Figure 4, Table 6), the highly permeable PSF-2 and PSF-4 supports were selected for the deposition of selective layers. The mechanical properties of the polymer in the most permeable support PSF-5 were insufficient for the reproducible production of composite membranes: it had a tensile strength of 1.0 MPa and a relative elongation of 6.7%. Composite membranes were also obtained using the porous PSF Ultrason^®^ S 6010. A synthesized laboratory sample of the polydecylmethylsiloxane–polymethyltrifluoroethylacrylate-siloxane F-containing copolymer (50F3) and a commercial sample of PDMS (Sylgard 184) were used as the selective layer materials. The F-containing polysiloxane 50F3 was chosen because the presence of polar components such as fluorinated groups in the siloxane structure increases the selectivity for CO_2_ [14]. A commercial PDMS sample was used for comparison.

#### 3.3.1. Morphology of Composite Membranes

**Figure 9:**
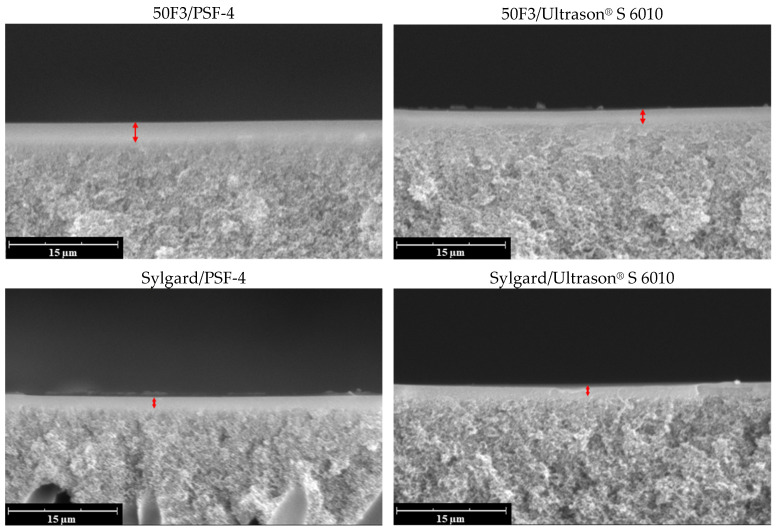
SEM microphotographs of cross-sections of composite membranes (the selective layer is marked with a red arrow).

SEM microphotographs of the cross-section of the composite membranes are shown in Figure 9. The thickness of the selective layer (marked with a red arrow) of each composite membrane was estimated from the SEM images. The results obtained are summarized in Table 7. It can be seen that the selective layer thickness varies between 1.9 and 2.5 μm in the case of 50F3, while the thickness of Sylgard 184 is 1.2–1.3 μm.

**Figure 10:**
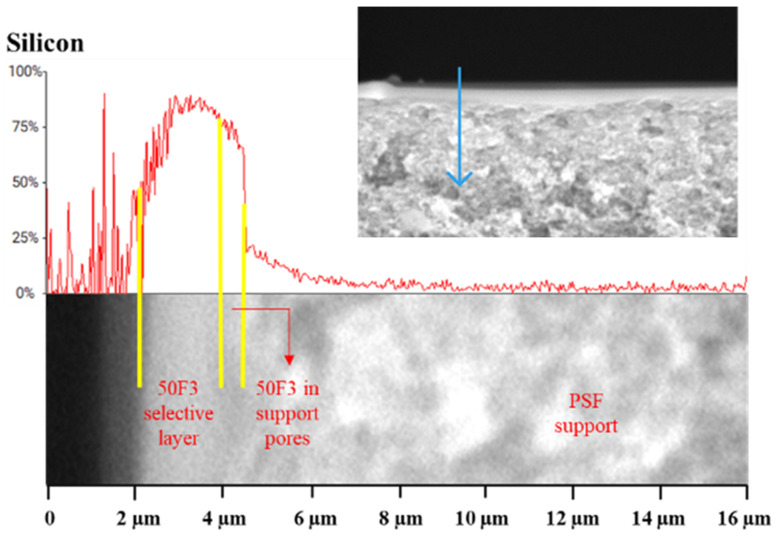
EDX profile of 50F3/Ultrason^®^ S 6010 composite membrane. The blue arrow indicates the depth of measurement.

To estimate the depth of penetration of the selective layer polymers into the pores of the support, EDX analysis of cross-sections of the composite membranes was performed. Silicon (Si), which is present in 50F3 and Sylgard 184 and absent in PSF, was chosen as the main chemical element for analyzing the distribution profiles. As an example, Figure 10 shows the EDX profile of a 50F3/Ultrason^®^ S 6010 composite membrane. It can be seen that the selective layer on the support surface consists of a silicon-containing material. Its thickness is approximately 2 μm, which is consistent with the SEM results (Table 7). The depth of penetration of the selective layer polymer into the porous support was evaluated according to a reported method [66]. EDX analysis showed that 50F3 penetrates into the support of commercial Ultrason^®^ S 6010 PSF to a depth of 0.5 μm. In the case of 50F3/PSF-2 and 50F3/PSF-4 composite membranes, the penetration depths are 0.8 and 0.6 μm, respectively. The depths of Sylgard 184 penetration into the pores of PSF-4 and commercial Ultrason^®^ S 6010 PSF supports were also estimated by the EDX profiles (Table 7) and amounted to 0.5 and 0.3 μm, respectively.

#### 3.3.2. Gas-Transport Properties of Composite Membranes

The implementation of the defect-free application of the selective layer was evaluated by comparison of individual gas selectivities of composite membranes and selective layer materials. PDMS is a widely used polymer whose gas-transport properties are well studied [67,68]. The 50F3 copolymer was studied for gas separation applications for the first time in this work. For this purpose, a 300 μm thick dense film of 50F3 was prepared, and the CO_2_ and CO permeabilities were measured (Table 8). It should be noted that the CO_2_ permeability is slightly lower for 50F3 (3040 Barrer) compared to PDMS (3200 Barrer). The CO_2_/CO selectivity of the 50F3 copolymer is 1.5 times higher than that of PDMS and equals 9.3, which is important for the possible practical application of this polymer for CO_2_/CO separation. Thus, as expected, the CO_2_ selectivity of polysiloxane with fluorine-containing groups is higher than that of PDMS, while the CO_2_ permeability is slightly smaller (by 4%). Table 8 also summarizes the gas-transport properties of high-permeability polymers used for CO_2_/CO separation [5]. The Pebax polymer demonstrates a 3-fold higher ideal CO_2_/CO selectivity compared to the 50F3 copolymer but a 35-fold lower CO_2_ permeability [69]. Glassy polytrimethylsilylpropyne (PTMSP) demonstrates high permeability values [68]. However, a significant drawback of PTMSP-based membranes lies in the problem of their physical aging, accompanied by a significant decrease in permeability with time [8].

Table 7 presents the values of gas permeances of the composite membranes developed for CO_2_ and CO individual gases. The similar values of ideal selectivities of the composite membranes (Table 7) and the selective layer materials, 50F3 and PDMS (Table 8), indicate the defect-free nature of the applied selective layer of the composite membrane.

The data presented in Table 7 also demonstrate the contribution of the support performance to the resulting gas-transport properties of the composite membranes. Of the composite membranes with the 50F3 selective layer, the membrane on the highly permeable PSF-2 support exhibits the highest permeance for CO_2_ (18,900 GPU). The CO_2_ and CO permeances of the 50F3/PSF-2 composite membrane are 258 and 28 GPU, respectively. This permeance value is 3.5 times higher than that of the 50F3/Ultrason^®^ S 6010 membrane, which is a consequence of the low (by an order of magnitude smaller) permeance of the PSF-2 and commercial Ultrason^®^ S 6010 PSF supports. It is noteworthy that both membranes have the same selective layer thickness (1.9 μm), and 50F3 copolymer penetrates into the PSF-2 support to a greater depth (0.8 μm) than into the Ultrason^®^ S 6010 PSF support (0.5 μm). The 50F3/PSF-4 composite membrane has a slightly lower permeance (25 and 232 GPU for CO and CO_2_, respectively) than the 50F3/PSF-2 membrane, despite the higher permeance of the PSF-4 support for CO_2_ (22,300 GPU). This is primarily due to the greater thickness of the 50F3 selective layer, which is 2.5 μm in the 50F3/PSF-4 membrane (Table 7). The selectivity values for all the membranes coincide and match the selectivity of the F-containing 50F3 polysiloxane (Table 8).

Similar trends are observed if PDMS Sylgard 184 is used as the selective layer. The permeance of the composite membrane based on the PSF-4 high-performance support (744 and 120 GPU for CO_2_ and CO, respectively) is 1.7 times higher than that of the composite membrane based on the lower-performance Ultrason^®^ S 6010 commercial PSF support (445 and 73 GPU for CO_2_ and CO, respectively). Thus, the enhanced gas permeance of the composite membranes based on highly permeable hydroxyl-terminated polysulfone supports is observed for both new F-containing 50F3 polysiloxane and the commercially available PDMS sample. The CO_2_/CO selectivity of the composite membranes 50F3/PSF-2 or 50F3/PSF-4 developed in this work (9.1–9.3) is 1.5 times higher than that of the composite membranes with a selective PDMS layer.

## 4. Conclusions

In this work, high-performance PSF supports and PSF-based composite membranes for efficient CO_2_/CO separation were developed. To create high-performance supports, PSF samples with terminal hydroxyl groups in a wide range of molecular weights (M_w_ = 76,000–122,000 g·mol^−1^) were synthesized for the first time by high-temperature polycondensation. The commercial PSF Ultrason^®^ S 6010, which, according to ^1^H NMR analysis, has predominantly terminal chlorine groups, was chosen as the polymer for comparison. Results of calculations of the Hansen solubility parameters showed that an increase in the ratio of terminal -OH groups improves the solubility of PSF in NMP. A similar dependence is observed for water. The kinetics of phase separation of casting solutions prepared from the PSF samples were studied. It was found that the coagulation rate depended significantly on the chemical composition of the terminal groups. The highest coagulation rate was demonstrated by PSF-based solutions with the highest ratio of the terminal hydroxyl groups (11.2 µm/s). The PSF/NMP solution with the commercial PSF Ultrason^®^ S 6010 demonstrates the smallest coagulation rate (3.9 µm/s).

Flat-sheet porous supports were prepared from PSF/NMP/PEG-400 solutions (21/49/30 wt. %) using the synthesized polymers and commercial PSF Ultrason^®^ S 6010, and their morphology and gas-transport properties were studied. The permeance of porous PSF supports was found to correlate with the coagulation rate of the casting solutions. It was shown that the use of synthesized PSF (M_w_ = 76,000 g·mol^−1^, M_w_/M_n_ = 3.0, (–OH):(–Cl) ratio as 4.7:1) enabled the porous support with CO_2_ permeance of 26,700 GPU to be obtained, while the support formed from commercial PSF Ultrason^®^ S 6010 (M_w_ = 68,000 g·mol^−1^, M_w_/M_n_ = 1.7, (–OH):(–Cl) ratio as 1:1.9) under the same conditions demonstrated 4300 GPU. The values of ideal selectivity (α (He/CO_2_) = 2.9–3.2) indicate the occurrence of a nearly-Knudsen gas flow regime for all supports made of the synthesized and commercial PSF samples. Moreover, the supports from the synthesized PSF are superior to the similar commercial porous PSF supports in the gas-transport properties and are not inferior to the properties of supports from other polymers.

The efficiency of the developed porous supports was estimated by making gas-separation composite membranes with selective layers from the synthesized polysiloxane containing fluorinated groups (50F3) and a commercial PDMS for CO_2_/CO separation (Sylgard 184). The 50F3 copolymer was investigated for the first time for gas separation tasks. It was found that the selectivity of the 50F3 material for the CO_2_/CO gas pair (9.3) was 1.5 times higher than that of PDMS (6.4), with a slight decrease in CO_2_ permeability (3040 Barrer for 50F3 and 3200 Barrer for PDMS). The similarity of the ideal selectivity values of the composite membranes and selective layer materials 50F3 and PDMS indicated that the applied selective layer of the composite membrane was defect free. The thickness of the selective layer was determined by the SEM method, and the depth of polymer penetration into the support pores was evaluated by EDX analysis. It was shown that, given the values of the selective layer thickness and depth of polymer penetration into the support pores are comparable, the performance of the composite membrane depends significantly on the support permeance. The permeance of composite membranes based on high-performance porous supports from synthesized PSF samples is 3.5 times higher than that of composite membrane based on supports from commercial PSF Ultrason^®^ S 6010. For example, the CO_2_ and CO permeances of the 50F3/PSF-2 composite membrane are 258 and 28 GPU, respectively. The improvement in gas permeance of composite membranes is reached by using the developed high-performance porous PSF supports, both with the 50F3 polysiloxane and with the commercial PDMS.

## Figures and Tables

**Table 1:** PSF structural groups and their group contributions [54].

Structural Group	*F_di_*, (MJ/m^3^)^1/2^·mol^−1^	*F_pi_*, (MJ/m^3^)^1/2^·mol^−1^	*E_hi_*, J/mol
–CH_3_	420	0	0
>C<	−70	0	0
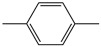	1270	110	0
–S–	440	0	0
–O–	100	400	3000
–OH	210	500	20,000
–Cl	450	550	400

**Table 2:** Molecular weight and structural parameters of PSF determined by the GPC method and calculated from ^1^H NMR spectra.

Sample	M_w_, g·mol^−1^	M_n_, g·mol^−1^	M_w_/M_n_	(-OH):(-Cl)	Number of Monomer Units	M_n(NMR)_, g·mol^−1^
PSF-1	122,000	40,000	3.1	2.4:1	35	24,000
PSF-2	111,000	39,000	2.8	3.0:1	33	22,000
PSF-3	100,000	35,000	2.9	2.4:1	32	21,000
PSF-4	97,000	35,000	2.8	3.1:1	32	21,000
PSF-5	76,000	25,000	3.0	4.7:1	21	16,000
Ultrason^®^ S 6010	68,000	39,000	1.7	1:1.9	37	23,000

**Table 3:** Hansen solubility parameters for PSF calculated by the group contribution method.

Sample	OH:Cl	M_n(NMR)_, g·mol^−1^	*δ_d_*, (MJ/m^3^)^1/2^	*δ_p_*, (MJ/m^3^)^1/2^	*δ_h_*, (MJ/m^3^)^1/2^	*δ*, (MJ/m^3^)^1/2^	∆*δ_P-S_*, (MJ/m^3^)^1/2^	∆*δ_P-NS_*, (MJ/m^3^)^1/2^
PSF-1	2.4: 1	24,000	18.76	4.64	5.99	20.23	7.79	38.28
PSF-2	3.0: 1	22,000	18.76	4.64	6.01	20.24	7.79	38.26
PSF-3	2.4: 1	21,000	18.76	4.64	6.00	20.24	7.79	38.27
PSF-4	3.1: 1	21,000	18.76	4.64	6.02	20.24	7.79	38.25
PSF-5	4.7: 1	16,000	18.75	4.64	6.16	20.28	7.77	38.12
Ultrason^®^ S 6010	1: 1.9	23,000	18.76	4.65	5.84	20.19	7.81	38.38
NMP [61]	-	-	18.0	12.3	7.2	23.0	-	-
Water [61]	-	-	15.5	16.0	42.4	47.9	-	-

**Table 4:** Kinetic characteristics of phase separation of PSF solutions.

Sample	PSF/NMP (21/79 wt. %)		PSF/NMP/PEG-400 (21/49/30 wt. %)		(-OH):(-Cl)
	Dynamic Viscosity, Pa·s	Coagulation Rate, µm/s	Dynamic Viscosity, Pa·s	Coagulation Rate, µm/s	
PSF-1	2.2	4.6	18.3	5.4	2.4: 1
PSF-2	1.7	9.0	16.1	10.6	3.0: 1
PSF-3	1.5	4.9	13.2	5.9	2.4: 1
PSF-4	1.3	9.4	10.8	11.1	3.1: 1
PSF-5	0.7	11.2	5.8	13.5	4.7: 1
Ultrason^®^ S 6010	3	3.9	18.1	4.5	1: 1.9

**Table 5:** Properties of porous PSF supports (1 GPU = 1 × 10^−6^ × (cm^3^(STP)/cm^2^∙cmHg∙s).

Support	L, μm	Permeance, GPU			α (He/N_2_)	α (He/CO_2_)	d_max_, nm
		He	CO_2_	N_2_			
PSF-1	120	39,900	12,500	16,000	2.5	3.2	48
PSF-2	110	56,400	18,900	23,300	2.4	3.0	73
PSF-3	110	41,400	14,300	17,800	2.3	2.9	89
PSF-4	120	67,500	22,300	29,100	2.3	3.0	60
PSF-5	110	82,700	26,700	33,100	2.5	3.1	53
Ultrason^®^ S 6010	120	13,200	4300	5400	2.4	3.0	62

**Table 6:** Comparison of the transport properties of the supports developed in this work with those of the commercial flat-sheet supports reported in the literature.

Support	Supplier of Polymer or Support	Permeance, GPU			α		Ref.
		He	CO_2_	N_2_	He/CO_2_	CO_2_/N_2_	
PSF		56,400	18,900	23,300	3.0	2.4	This work
		67,500	22,300	29,100	3.0	2.3	
		82,700	26,700	33,100	2.5	3.1	
PSF Ultrason^®^ S 6010	BASF, Ludwigshafen, Germany	13,200	4300	5400	2.4	3.0	This work
PSF	Alpha Laval, Koldin, Denmark	-	100, 1000	-	-	-	[21]
PES		-	500, 3000	-	-	-	
PAN	Fujifilm, Tokyo, Japan	-	10,000	-	-	-	
PVDF		-	1000	-	-	-	
PSF	Vontron Technology, Beijing, China	-	16,900	20,700	-	-	[63]
PES Ultrason^®^ E 7020P	BASF, Ludwigshafen, Germany	-	9200	~10,200	-	-	[64]
PAN/PPS	Sanofi-Aventis, Paris, France	-	-	31,500	-	-	[65]

**Table 7:** Transport properties of composite membranes.

Membrane	Selective Layer Thickness, µm	The Depth of Polymer Penetration into the Support Pores, µm	Permeance, GPU		Selectivity α
			CO_2_	CO	CO_2_/CO
50F3/PSF-2	1.9	0.8	258	28	9.2
50F3/PSF-4	2.5	0.6	232	25	9.3
50F3/Ultrason® S 6010	1.9	0.5	75	8.2	9.1
PDMS/PSF-4	1.3	0.5	744	120	6.2
PDMS/Ultrason® S 6010	1.2	0.3	445	73	6.2

**Table 8:** Gas-transport properties of the selective layer materials used in this work and of other high permeability polymers for CO_2_/CO separation [5].

Polymer	Permeability, Barrer		Selectivity α	Ref.
	CO_2_	CO	CO_2_/CO	
50F3	3040	326	9.3	This work
PDMS	3200	500	6.4	[68]
Pebax	87.5	2.9	30	[69]
PTMSP	18,200	5400	3.4	[68]

## Data Availability

Data is contained within the article or Supplementary Information.

## Supplementary Materials

The following supporting information can be downloaded at https://www.mdpi.com/article/10.3390/polym16243453/s1, Figure S1: DSC spectra of the PSF samples synthesized; Figure S2: TGA spectra of the PSF samples synthesized; Figure S3: Dependence of the dynamic viscosity of casting solutions of PSF/NMP (21/79 wt. %) and PSF/NMP/PEG-400 (21/49/30 wt. %) on the molecular weight of the polymer; Table S1: Thermal properties of the PSF synthesized.
