# Supplementary material for: High-Performance Porous Supports Based on Hydroxyl-Terminated Polysulfone and CO2/CO-Selective Composite Membranes
Source: Polymers (Basel). 2024 Dec 10;16(24):3453. doi: 10.3390/polym16243453 (PMC11680076; doi:10.3390/polym16243453)
Supplement: Supplementary file 1 [file polymers-16-03453-s001.zip › polymers-3328676-supplementary.pdf]

1. Differential scanning calorimetry

The DSC method was employed to determine the glass transition temperature T_g of the synthesized PSF samples. The DSC spectra obtained are presented in Figure S1. Based on Figure S1, it is noted that an increase in the M_w of polymers leads to an increase in the glass transition temperature from 186.2 to 191.4 °C, regardless of the proportion of hydroxyl and chlorine terminal groups. A similar dependence of T_g on M_w was demonstrated in [1], where the glass transition temperature of the PSF did not depend on the conditions under which the polymer was synthesized. Similar correlations were also observed for another polymer from the polysulfone family, polyphenylene sulfone [2]. The glass transition temperatures of the commercial PSF ULTRASON® S 6010, Udel® P 3500, and Sigma-Aldrich are 187 [3,4], 186 [5], and 187 °C [6], respectively.

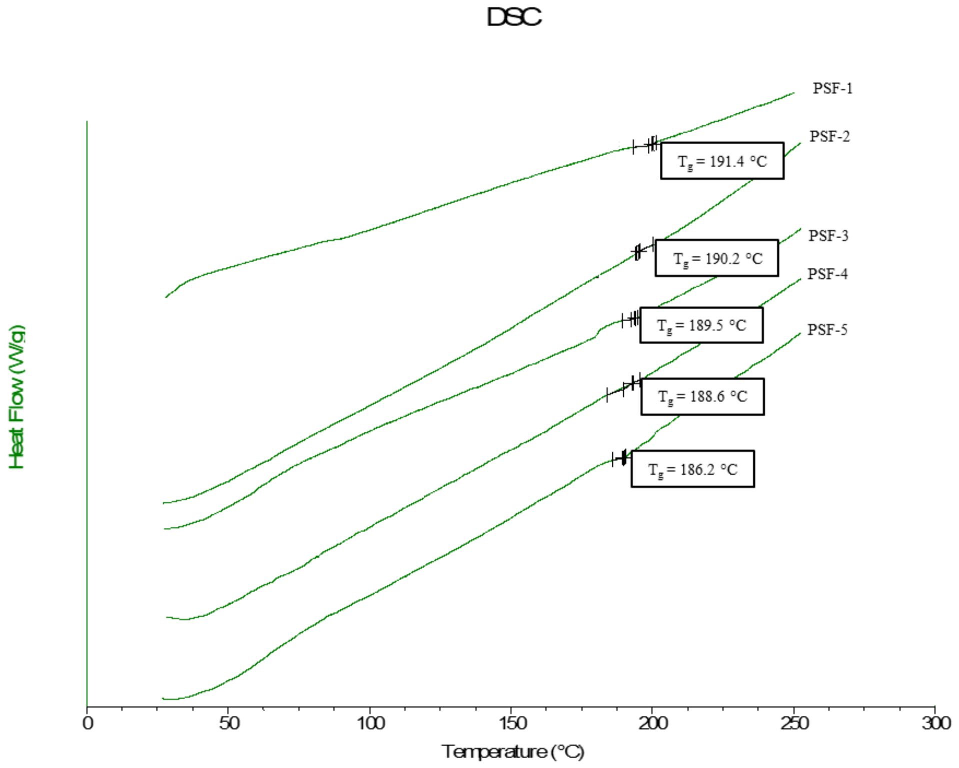

Figure S1. DSC spectra of the PSF samples synthesized.

2. Thermogravimetric analysis

The thermal stability of the synthesized samples was assessed using the TGA technique in air over the temperature range of 30-750 °C (Figure S2). Analysis of the thermograms of each sample revealed that at temperatures lower than 170 °C, there is no polymer mass loss, indicating that the solvent (DMAc) is completely removed from the synthesized PSF samples. Table S1 presents the results of determining the temperatures at which a 2% ($T_{2\%}$), 5% ($T_{5\%}$), and 10 % ($T_{10\%}$) loss of polymer mass occurs. It can be seen from Table S1 that as the molecular weight

increases, the temperatures of 2% and 5% mass loss of the polymer increase significantly (443 and 480 °C, respectively, for PSF-1 with a molecular weight of 122 000 g·mol^{-1}, and 425 and 465 °C, respectively, for PSF-5 with a molecular weight of 76 000 g·mol^{-1}). At the same time, the 10% loss of the polymer mass occurs within a narrow temperature range between 490 and 496 °C. For commercial samples of PSF from Sigma-Aldrich and Solvay (Udel® P 3500 brand) companies, the 10 % loss of the polymer mass occurs at ~510 °C [7] and ~530 °C [8], respectively. The higher values for the commercial polymers can be primarily attributed to the fact that their TGA analysis was carried out in a nitrogen atmosphere where there are thermal oxidative degradation processes do not occur [7,8].

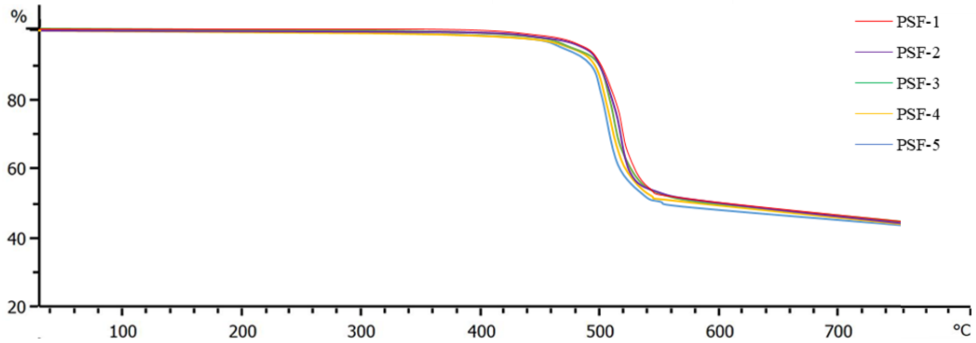

Figure S2. TGA spectra of the PSF samples synthesized

Table S1. Thermal properties of the PSF synthesized.

Polymer	M_w, g·mol^{-1}	T_g, °C	$T_{2\%}$, °C	$T_{5\%}$, °C	$T_{10\%}$, °C
PSF-1	122 000	191.4	443	480	496
PSF-2	111 000	190.2	443	478	496
PSF-3	100 000	189.5	432	470	496
PSF-4	97 000	188.6	428	472	494
PSF-5	76 000	186.2	425	465	490

3. Dynamic viscosity of PSF solutions

The dynamic viscosity of the PSF/NMP (21/79 wt.%) and PSF/NMP/PEG-400 (21/49/30 wt.%) polymer solutions was studied. The results obtained are presented in Figure S3. As can be

seen, with an increase in the molecular weight of the synthesized PSF from 76 000 to 122 000 g·mol^{-1}, the dynamic viscosity of two-component solutions varied from 0.7 to 2.2 Pa·s, while that of three-component solutions ranged from 5.8 to 18.3 Pa·s.

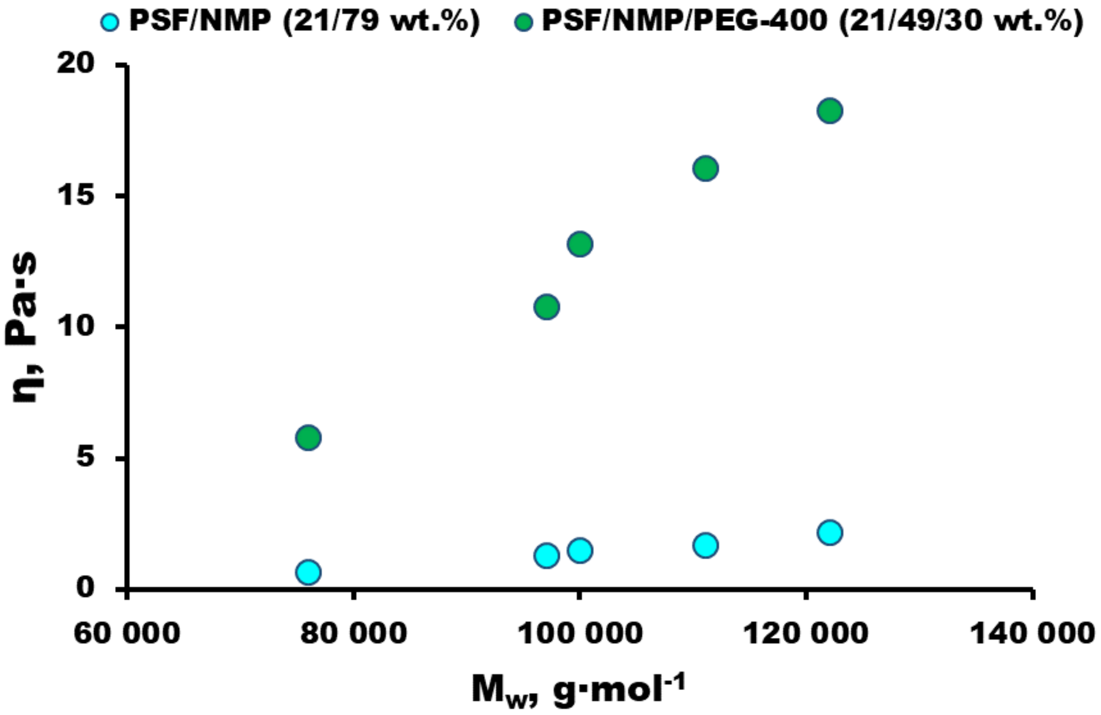

Figure S3. Dependence of the dynamic viscosity of casting solutions of PSF/NMP (21/79 wt, %) and PSF/NMP/PEG-400 (21/49/30 wt. %) on the molecular weight of the polymer.

References

[1] D. Matveev, A. Raeva, I. Borisov, V. Vasilevsky, Y. Matveeva, A. Zhansitov, S. Khashirova, V. Volkov, Effect of Molecular Weight and Chemical Structure of Terminal Groups on the Properties of Porous Hollow Fiber Polysulfone Membranes, Membranes 13 (2023) 412. https://doi.org/10.3390/membranes13040412.

[2] A. Zhansitov, Z. Kurdanova, K. Shakhmurzova, A. Slonov, I. Borisov, S. Khashirova, Effect of Solvent and Monomer Ratio on the Properties of Polyphenylene Sulphone, Polymers 15 (2023) 2279. https://doi.org/10.3390/polym15102279.

[3] Y. Jia, S. Sun, S. Li, Z. Wang, F. Wen, C. Li, H. Matsuyama, S. Hu, Improved Performance of Polysulfone Ultrafiltration Membrane Using TCPP by Post-Modification Method, Membranes 10 (2020) 66. https://doi.org/10.3390/membranes10040066.

[4] S. Basu, A. Cano-Odena, I.F.J. Vankelecom, Asymmetric membrane based on Matrimid® and polysulphone blends for enhanced permeance and stability in binary gas (CO2/CH4) mixture separations, Separation and Purification Technology 75 (2010) 15–21. https://doi.org/10.1016/j.seppur.2010.07.004.

[5] M.O. Midda, A.K. Suresh, Some mechanistic insights into the action of facilitating agents on gas permeation through glassy polymeric membranes, AIChE Journal 64 (2018) 186–199. https://doi.org/10.1002/aic.15873.

[6] G.D. Vilakati, E.M.V. Hoek, B.B. Mamba, Investigating the structure and water permeation of membranes modified with natural and synthetic additives using tensile, porosity, and glass transition temperature studies, Journal of Applied Polymer Science 131 (2014). https://doi.org/10.1002/app.40616.

[7] A.M. Martos, J.-Y. Sanchez, A. Várez, B. Levenfeld, Electrochemical and structural characterization of sulfonated polysulfone, Polymer Testing 45 (2015) 185–193. https://doi.org/10.1016/j.polymertesting.2015.06.004.

[8] O. Petreus, E. Avram, D. Serbezeanu, Synthesis and characterization of phosphorus-containing polysulfone, Polymer Engineering & Science 50 (2010) 48–56. https://doi.org/10.1002/pen.21508.
